# Common and Specific Alterations of Amygdala Subregions in Major Depressive Disorder With and Without Anxiety: A Combined Structural and Resting-State Functional MRI Study

**DOI:** 10.3389/fnhum.2021.634113

**Published:** 2021-02-15

**Authors:** Yao Yao Li, Xiao kang Ni, Ya feng You, Yan hua Qing, Pei rong Wang, Jia shu Yao, Ke ming Ren, Lei Zhang, Zhi wei Liu, Tie jun Song, Jinhui Wang, Yu-Feng Zang, Yue di Shen, Wei Chen

**Affiliations:** ^1^Department of Psychiatry, Hangzhou Seventh People's Hospital, Hangzhou, China; ^2^Department of Psychiatry, Sir Run Run Shaw Hospital, Zhejiang University School of Medicine, Hangzhou, China; ^3^Zhejiang Academy of Traditional Chinese Medicine, Hangzhou, China; ^4^Clinical Laboratory, Sir Run Run Shaw Hospital, Zhejiang University School of Medicine, Hangzhou, China; ^5^Guangdong Key Laboratory of Mental Health and Cognitive Science, Center for Studies of Psychological Application, Institute for Brain Research and Rehabilitation, South China Normal University, Guangzhou, China; ^6^Zhejiang Key Laboratory for Research in Assessment of Cognitive Impairments, Hangzhou, China; ^7^Department of Diagnostics, School of Medicine, Hangzhou Normal University, Hangzhou, China; ^8^Department of Psychology and Behavioral Sciences, Zhejiang University, Hangzhou, China; ^9^Key Laboratory of Medical Neurobiology of Zhejiang Province, Hangzhou, China

**Keywords:** major depressive disorder, multimodal MRI, amygdala subregion, anxiety, functional connectivity

## Abstract

Anxious major depressive disorder is a common subtype of major depressive disorder; however, its unique neural mechanism is not well-understood currently. Using multimodal MRI data, this study examined common and specific alterations of amygdala subregions between patients with and without anxiety. No alterations were observed in the gray matter volume or intra-region functional integration in either patient group. Compared with the controls, both patient groups showed decreased functional connectivity between the left superficial amygdala and the left putamen, and between the right superficial amygdala and the bilateral anterior cingulate cortex and medial orbitofrontal cortex, while only patients with anxiety exhibited decreased activity in the bilateral laterobasal and superficial amygdala. Moreover, the decreased activity correlated negatively with the Hamilton depression scale scores in the patients with anxiety. These findings provided insights into the pathophysiologic processes of anxious major depressive disorder and may help to develop new and effective treatment programs.

## Introduction

Major depressive disorder (MDD) is a common mental disorder that affects more than 300 million people globally (Gaspersz et al., 2017a). MDD is becoming the leading cause of disability worldwide and contributes significantly to the global disease burden. MDD is a clinically heterogeneous disease with multiple subtypes, among which anxious MDD (AMDD) is one of the most common, with a prevalence of 40–60% (Gaspersz et al., 2017a). Compared with non-anxious MDD (nAMDD), patients with AMDD often suffer from more severe depressive illness (Wiethoff et al., 2010; Goldberg et al., 2014), greater functional impairment (Rao and Zisook, 2009; Goldberg and Fawcett, 2012), reduced response to antidepressant treatment (Fava et al., 2008; Ionescu et al., 2014), and a higher risk of suicide (Seo et al., 2011; Gaspersz et al., 2017b). Thus, it is clinically significant to explore the unique neural mechanism underlying this specific MDD subtype, which could help to develop new and effective treatment programs for AMDD.

The amygdala, which is located in the medial part of the anterior temporal lobe, is one of the most important structures in the limbic system (Ongur and Price, 2000). It plays a vital role in emotion processing and regulation (Phillips and Swartz, 2014). Mounting evidence from multimodal MRI studies has indicated that the amygdala is widely implicated in the pathophysiology of depression, as characterized by structural and functional alterations in patients (Savitz and Drevets, 2009; Licznerski and Duman, 2013; Fonseka et al., 2018). With respect to AMDD, a previous study used diffusion and structural MRI and showed no significant differences in volume or white matter integrity of the amygdala between patients with AMDD and nAMDD (Delaparte et al., 2017). Analogously, analyses of functional MRI (fMRI) revealed that patients with AMDD and those with nAMDD exhibited common patterns of abnormal activation and connectivity of the amygdala compared with controls (Etkin and Schatzberg, 2011; van Tol et al., 2012). However, it is worth noting that all the above studies treated the amygdala as a unified structure. Recent evidence indicates anatomical and functional heterogeneity of the human amygdala, which can be divided into three major subdivisions: The laterobasal amygdala (LBA), the superficial amygdala (SFA), and the centromedial amygdala (CMA) (Amunts et al., 2005; Ball et al., 2007). These subregions exhibit different connectivity patterns in healthy individuals (Li et al., 2012) and show unique alterations in MDD (Wang et al., 2017) and anxiety disorder (Qin et al., 2014). However, whether and how the amygdala subregions are differentially involved in AMDD are largely unknown.

In the present study, we investigated common and specific structural and functional brain alterations of amygdala subregions between patients with AMDD and those with nAMDD by combining structural MRI and resting-state fMRI (R-fMRI). Specifically, for each subregion of the amygdala, we compared 26 patients with AMDD and 23 patients with nAMDD vs. 30 healthy controls (HCs) in terms of regional gray matter volume (GMV) using structural MRI, and amplitude of low frequency fluctuation (ALFF), cross-correlation coefficients of spontaneous low-frequency (COSLOF), and seed-based functional connectivity (sFC) using R-fMRI. We hypothesized that compared with the HCs, patients with AMDD and nAMDD had common and specific alterations in the amygdala that were dependent on the subregions.

## Materials and Methods

### Participants

All patients included in this study were screened from an ongoing follow-up project that aims to explore the relationships between baseline brain architecture and clinical outcomes of patients with MDD after antidepressant treatment using multimodal MRI data. According to the aim of the current study, 49 patients with MDD (26 AMDD and 23 nAMDD) were selected. MDD was diagnosed according to the Diagnostic and Statistical Manual of Mental Disorders, 4th Edition, Text Revision (DSM-IV-TR) criteria, using the Structured Clinical Interview for DSM-V (SCID)-I. Exclusion criteria for MDD included (1) patients who could not undergo an MRI examination because of claustrophobia, metallic implants, or other contraindication for MRI; (2) patients with severe suicidal tendency; (3) pregnant or lactating women; (4) any severe physical diseases as assessed by personal history; (5) a history of organic brain disorders, neurological disorders, other psychiatric disorders, cardiovascular diseases, head trauma, or loss of consciousness; and (6) a history of substance abuse, including tobacco, alcohol, or other psychoactive substances. The patients were recruited from outpatients and inpatients of the Sir Run Run Shaw Hospital, School of Medicine, Zhejiang University, Hangzhou, China. All patients were free of psychotropic medications for at least 4 weeks before the MRI scan and had a Hamilton depression rating (HAMD) score ≥ 14. We chose a HAMD ≥ 14 instead of 18 as in our previous studies (Shen et al., 2015; Sheng et al., 2018) to maximize the sample size of the current study. Out of the 49 patients, 26 were categorized as having AMDD in terms a HAMD anxiety/somatization factor score ≥ 7 (Fava et al., 2008). In addition, a cohort of 30 HCs was enrolled using community recruitment via an advertisement. All HCs had no lifetime history of psychiatric or neurological illness and no lifetime history of substance abuse. All participants were right-handed Han Chinese individuals, aged 18–60 years old, and had a mood Disorder Questionnaire (MDQ) score < 7. Demographic and clinical characteristics of all the participants are summarized in Table 1. This study was approved by the Ethics Committee of the Sir Run Run Shaw Hospital, School of Medicine, Zhejiang University, and the Affiliated Hospital of Hangzhou Normal University. All participants gave written informed consent.

**Table 1:** Demographics and clinical measures of all participants.

	**HCs (*n* = 30)**	**AMDD (*n* = 26)**	**NAMDD (*n* = 23)**	***P*-value**
Age (years)	39.233 ± 12.484	41.308 ± 10.884	36 ± 10.135	0.264
Gender (M/F)	12/18	11/15	15/8	0.147
HAMD		22.808 ± 4.578	18.044 ± 2.931	<0.001
Anxiety/somatization factor scores		8.769 ± 1.704	5.348 ± 0.714	<0.001
Others		14.039 ± 3.893	12.696 ± 2.883	0.181
Duration of illness (years)		0.2 – 13	0.1 – 16	0.277
Age of onset (years)		37.654 ± 12.614	31.696 ± 9.665	0.073

*HCs, healthy controls; AMDD, anxious major depressive disorder; NAMDD, non-anxious major depressive disorder; M, male; F, female; HAMD, Hamilton Rating Scale for Depression*.

### MRI Data Acquisition

All MRI data were acquired on a 3.0 T MR scanner (GE Discovery MR750, GE Medical Systems, Milwaukee, WI, USA) equipped with an eight-channel head coil array in the Center for Cognition and Brain Disorders, Hangzhou Normal University, China. During the scan, all participants were instructed to lie quietly in the scanner with their eyes closed, to keep their head still, but awake, and to try not to think of anything systematically. High-resolution T1-weighted images were acquired with a three-dimensional spoiled gradient-recalled sequence with the following parameters: 176 axial slices; repetition time (TR) = 8.1 ms; echo time (TE) = 3.1 ms; ip angle (FA) = 8°; field of view (FOV) = 250 × 250 mm^2^; matrix = 256 × 256; slice thickness = 1.0 mm; and no gap. The R-fMRI images were acquired axially using a single-shot, gradient-recalled echo planar imaging sequence parallel to the line of the anterior–posterior commissure: 37 slices; TR = 2,000 ms; TE = 30 ms; FA = 90°; FOV = 220 × 220 mm^2^; matrix = 128 × 128; slice thickness = 3.2 mm; and no gap. The scan lasted for 368 s in total and included 184 volumes for each participant.

### Definition of Amygdala Subregions

The amygdala subregions were determined using stereotaxic, probabilistic maps of cytoarchitectonic boundaries (Amunts et al., 2005) and implemented in FSL's Juelich histological atlas (https://fsl.fmrib.ox.ac.uk/fsl/fslwiki/). All amygdala subregions were created in the standard Montreal Neurological Institute (MNI) space by selecting only voxels with a probability of at least 50% of belonging to each subdivision.

### Structural MRI Data Analysis

Structural images were processed by using the cat toolbox (http://www.neuro.uni-jena.de/cat/) for the SPM12 (http://www.fil.ion.ucl.ac.uk/spm/software/spm12/). Briefly, individual structural images were first segmented into GM, white matter, and cerebrospinal fluid on the basis of an adaptive Maximum A Posterior technique. The resultant GM maps were then normalized to the MNI space by using a high-dimensional “DARTEL” approach and modulated to compensate for spatial normalization effects. Finally, for each amygdala subregion, the mean GMV was obtained for each participant by averaging the GMV values across the voxels within the subregion.

### R-fMRI Data Analysis

All R-fMRI data preprocessing was implemented using GRETNA (Wang et al., 2015b) based on the SPM12 software (http://www.fil.ion.ucl.ac.uk/spm/software/spm12/). After discarding the first five volumes to allow for magnetic saturation, we conducted slice timing (Sinc interpolation), head motion correction (six-parameter rigid-body transformation), spatial normalization (via tissue segmentation of individual structural images using the unified segment) and resampling (3 × 3 × 3 mm^3^), and smoothing (Gaussian kernel with a 6-mm full width at half maximum). No patients were excluded according to the head motion criteria of > 2.5 mm in displacement or > 2.5° in rotation in any direction (the maximum head motion was 2.275 mm in translation and 1.617° in rotation over all participants). To ensure functional specificity of the amygdala subregions, all regions of interest (ROIs) were masked out before spatial smoothing. The smoothed images subsequently underwent removal of linear trends and band-pass filtering (0.01–0.08 Hz). Finally, the white matter signals, cerebrospinal fluid signals, and 24-parameter head motion profiles (Friston et al., 1996) were regressed out from each voxel's time series. The white matter signals and cerebrospinal fluid signals were obtained by averaging signals in masks derived from the corresponding tissue probability maps in the SPM12 toolbox (thres = 0.8) (Wang et al., 2015a). The high threshold was chosen to ensure that the resultant mask was “entirely” from white matter or CSF. Notably, all the nuisance signals were also band-pass filtered (0.01–0.08 Hz) (Hallquist et al., 2013).

After preprocessing, three measures were used to comprehensively characterize the functional architecture of each amygdala subregions, including ALFF, COSLOF, and sFC. (1) ALFF. For each voxel within a given amygdala subregion, the time series was first converted to the frequency domain using a fast Fourier transformation. The square root of the power spectrum was then computed and summed across a predefined frequency interval (0.01–0.08 Hz). The ALFF for the given amygdala subregion was finally calculated as the mean summed square root across voxels within the subregion. ALFF measures the strength or intensity of low-frequency oscillations embedded in spontaneous neural activity (Zang et al., 2007). (2) COSLOF. For any pair of voxels within a given amygdala subregion, the Pearson correlation coefficient was first computed between their time series. This resulted in a correlation matrix with a dimensionality of *n* × *n* (*n* = the number of voxels in the given subregion). The COSLOF for the given amygdala subregion was then computed as the mean of all the elements in the upper triangular portion of the correlation matrix. COSLOF reflects the overall functional integration within a given region (Li et al., 2002). (3) sFC. For each voxel within a given amygdala subregion, the time series was first extracted and then correlated with the time series of each voxel over the entire brain. This resulted in six whole-brain FC maps for each participant. The functional connectivity maps further underwent a Fisher's r-to-z transformation to improve the normality. Notably, the sFC analysis was restricted in a gray matter mask that was obtained by thresholding the gray matter tissue probability map in the SPM12 toolbox (thres = 0.4). The relatively loose threshold was chosen to ensure sufficient search space for gray matter.

### Statistical Analysis

For the demographic data, one-way analysis of variance (ANOVA) and chi-square tests were used to compare age and gender data among the three groups, respectively. For the clinical data, two-sample *t*-tests were used to compare HAMD anxiety/somatization factor scores, HAMD without anxiety/somatization factor scores, age of onset, and duration of illness between the two patient groups. For multimodal MRI-based GMV, ALFF, and COSLOF of each amygdala subregion, one-way ANOVA was used to infer differences among the three groups, followed by a Bonferroni method to correct for multiple comparisons across subregions. For the whole-brain sFC maps, a voxel-wise F-test was performed to infer differences among the three groups. A cluster-level family-wise error rate procedure was used to determine significant clusters by combining a voxel-level *P* < 0.001 and an extend-level *P* < 0.05. *Post-hoc* pair-wise comparisons were further performed with two-sample *t*-tests when significant main effects were observed. For the sFC, the *post-hoc* comparisons were conducted on the intra-cluster mean FC strength. Finally, non-parametric Spearman correlations were used to test the relationships between multimodal MRI-based amygdala alterations and clinical variables (HAMD anxiety/somatization factor scores, HAMD without anxiety/somatization factor scores, duration of illness, and age of onset) in each patient group. Again, the Bonferroni method was used to correct for multiple comparisons.

## Results

### Demographic and Clinical Characteristics

As shown in Table 1, there were no significant differences in age or gender among the three groups (*P* > 0.05). Both patient groups did not differ significantly in their HAMD without anxiety/somatization factor scores, age of onset, or duration of illness (*P* > 0.05). As expected, the HAMD scores were significantly higher in the AMDD group than the nAMDD group (*P* < 0.001) because of their higher anxiety/somatization factor scores (*P* < 0.001).

### Structural GMV of Amygdala Subregions

No significant differences were observed in the GMV for any amygdala subregion among the three groups (*P* > 0.05, Bonferroni corrected).

### Functional ALFF of Amygdala Subregions

**Figure 1:**
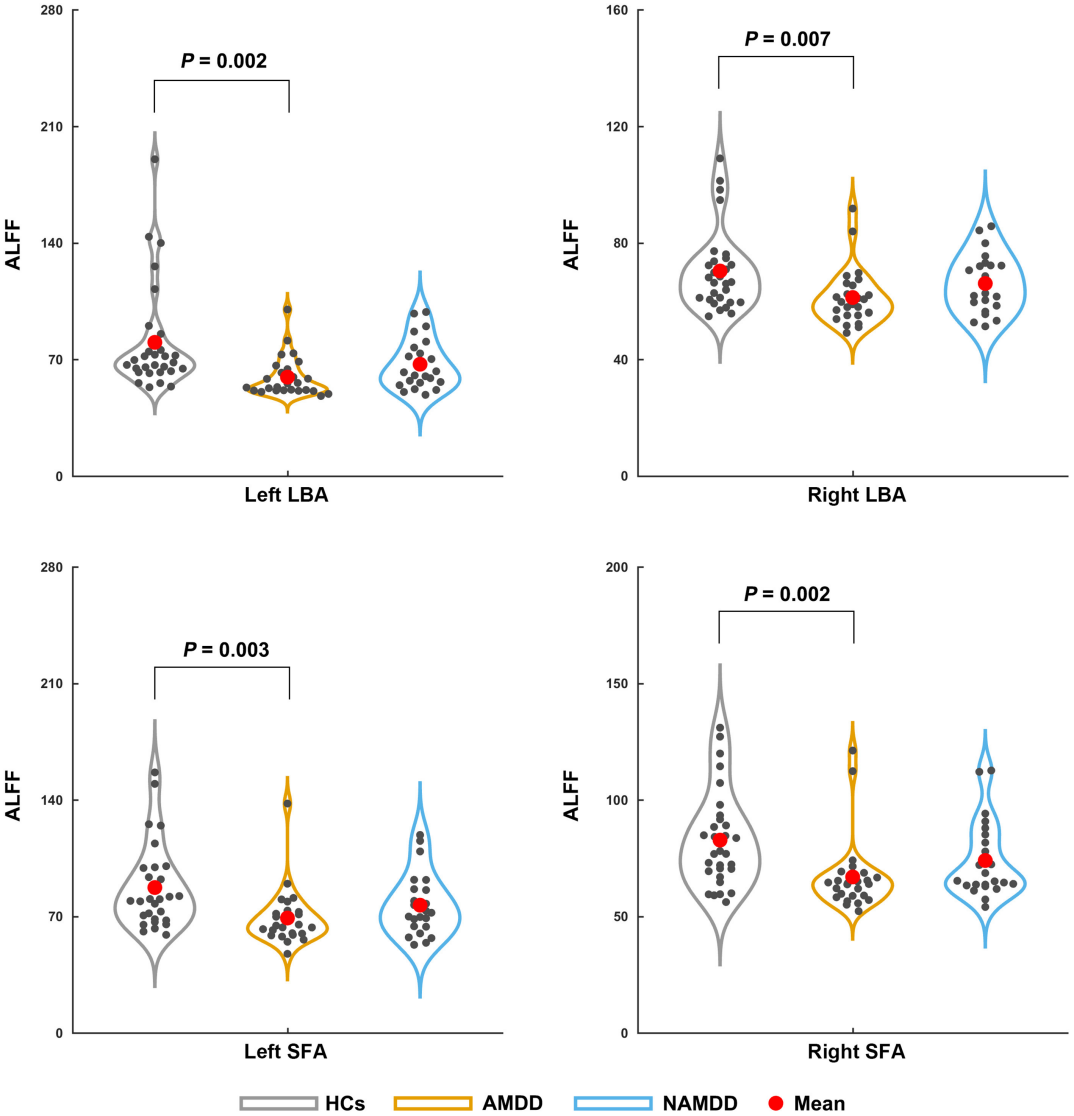
Differences in the ALFF of amygdala subregions among the groups. Significant ALFF decreases in the bilateral LBA and SFA were observed in patients with AMDD but not in those with nAMDD compared with the HCs. ALFF, amplitude of low frequency fluctuation; LBA, laterobasal amygdala; SFA, superficial amygdala; AMDD, anxious major depressive disorder; nAMDD, non-anxious major depressive disorder; HCs, healthy controls.

Significant main effects (*P* < 0.05, Bonferroni corrected) were found for the ALFF of the bilateral LBA (left: *P* = 0.002; right: *P* = 0.007) and SFA (left: *P* = 0.003; right: *P* = 0.002), but not the bilateral CMA (*P* > 0.05), among the three groups (Figure 1). *Post-hoc* comparisons revealed a common pattern of HCs > nAMDD > AMDD that were shared by the four subregions, while only the differences between the patients with AMDD and HCs were significant (*P* < 0.05, Bonferroni corrected).

### Functional COSLOF of Amygdala Subregions

No significant differences were observed in the COSLOF for any amygdala subregion among the three groups (*P* > 0.05, Bonferroni corrected).

### Functional sFC of Amygdala Subregions

**Figure 2:**
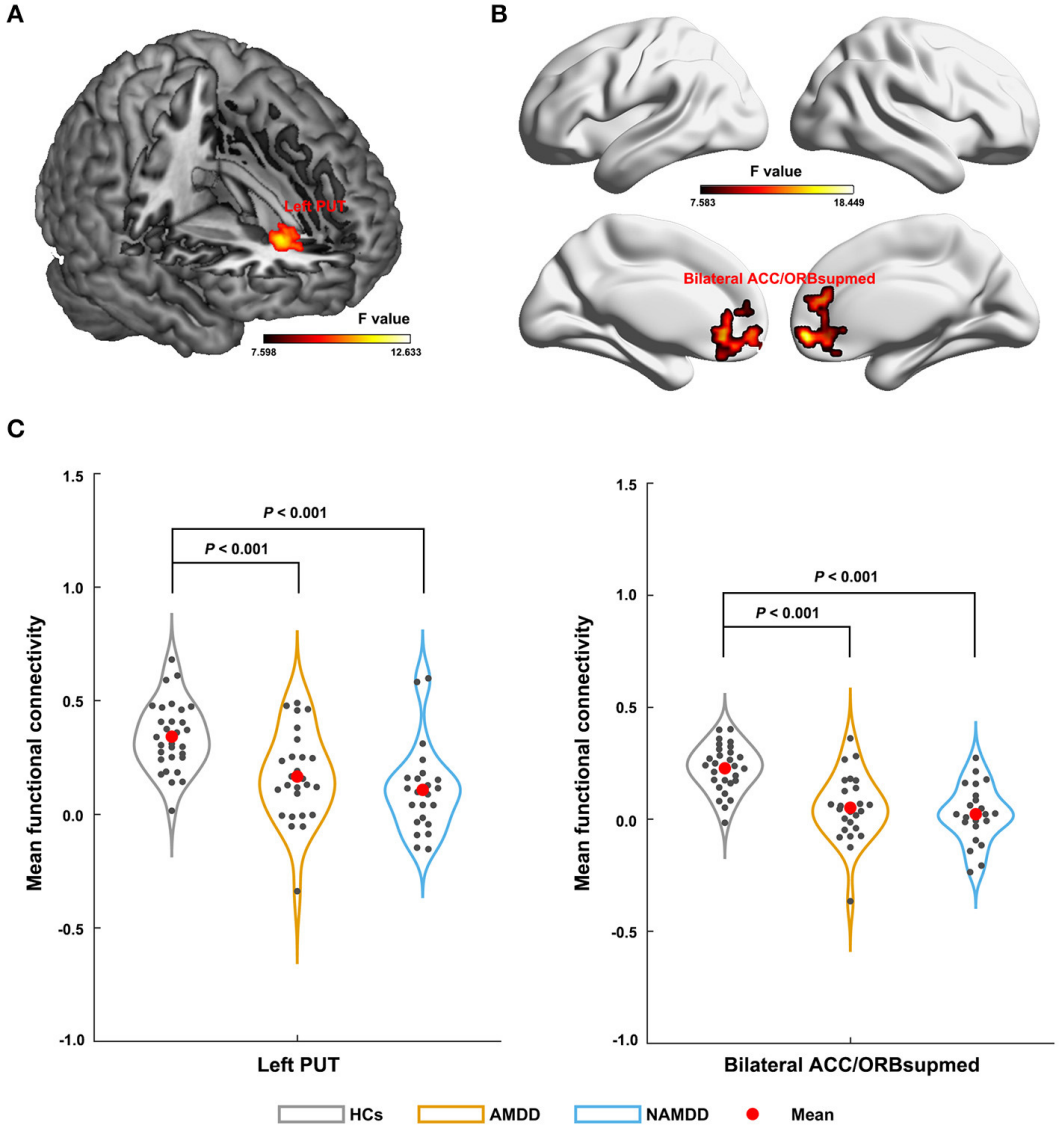
Differences in sFC of amygdala subregions among groups. The left SFA-based sFC **(A)**; the right SFA-based sFC **(B)**; Significant sFC decreases were observed in both the patient groups compared with that in the HCs between the left SFA and the left putamen, and between the right SFA and the bilateral anterior cingulate cortex and medial orbital parts of the bilateral superior frontal gyri **(C)**. PUT, putamen; ACC, anterior cingulate cortex; ORBsupmed, medial orbital part of the superior frontal gyrus; AMDD, anxious major depressive disorder; nAMDD, non-anxious major depressive disorder; HCs, healthy controls.

Significant main effects (*P* < 0.05, FWE corrected) were observed for the left SFA-based sFC in a cluster that primarily embraced the left putamen (Figure 2A) and for the right SFA-based sFC in a cluster that was predominantly involved in the bilateral anterior cingulate cortex (ACC) and medial orbital parts of the bilateral superior frontal gyri (ORBsupmed) (Figure 2B). Further *post-hoc* comparisons revealed that both patient groups showed significantly decreased FC compared with those of the HCs, but with no significant differences between them (Figure 2C). For the bilateral CMA and LBA, no regions were found to show significant main effects among the three groups (*P* > 0.05, FWE corrected).

### Relationships Between Functional Characteristics of Amygdala Subregions and Clinical Variables

**Figure 3:**
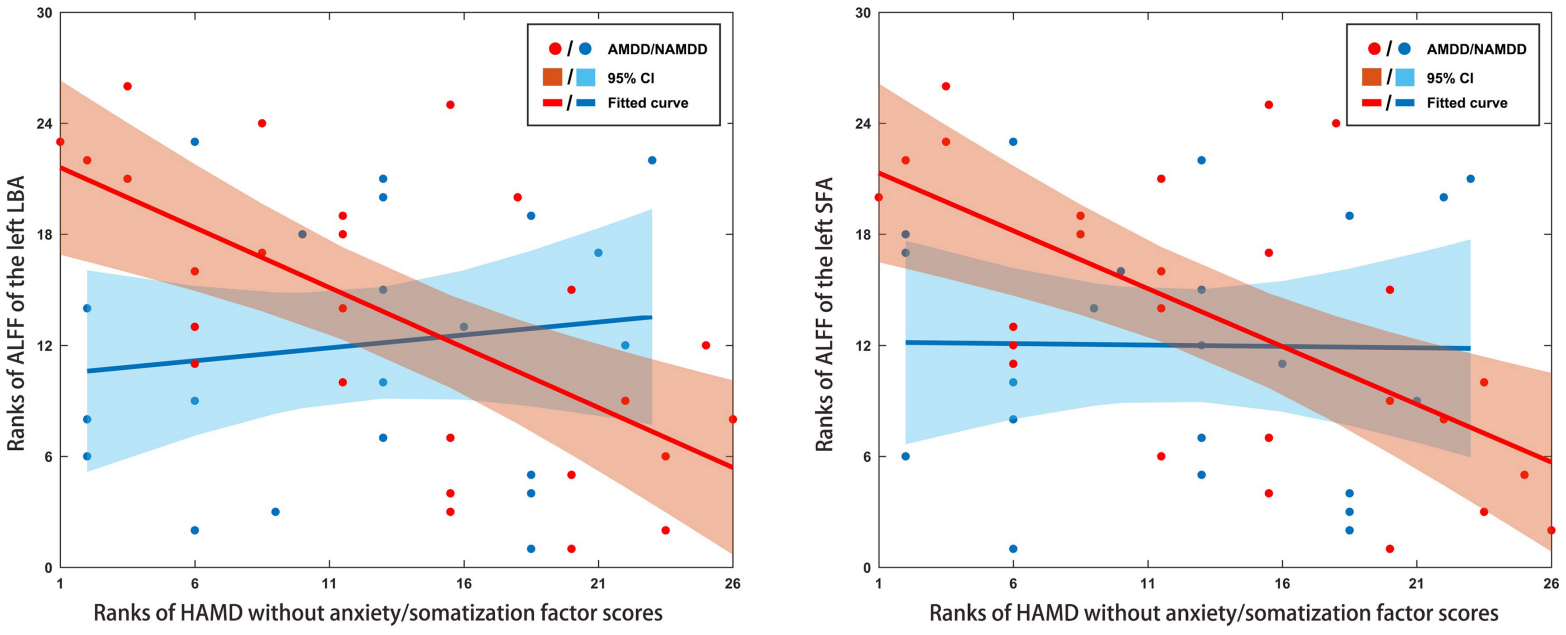
Scatter plots of relationships between altered ALFF of amygdala subregions and clinical variables in patients. Significant negative correlations were observed for the ALFF of the left LBA and SFA with HAMD without anxiety/somatization factor scores in the AMDD group, but not in the nAMDD group. ALFF, amplitude of low frequency fluctuation; LBA, laterobasal amygdala; SFA, superficial amygdala; AMDD, anxious major depressive disorder; nAMDD, non-anxious major depressive disorder; HAMD, Hamilton Rating Scale for Depression.

Significant negative correlations were observed for the ALFF of the left LBA and SFA with HAMD without anxiety/somatization factor scores in the patients with AMDD (*r* = −0.645 and −0.622, respectively, *P* < 0.05, Bonferroni corrected) rather than in those with nAMDD (*P* > 0.05) (Figure 3). No significant correlations were found between ALFF and other clinical variables or between sFC and any clinical variables.

## Discussion

This study investigated the structural and functional alterations of amygdala subregions in patients with AMDD and nAMDD vs. HCs. Our results showed common and specific amygdala alterations between patients with AMDD and nAMDD that were dependent on different amygdala subdivisions. These findings provide new insights into the unique neural mechanism underlying AMDD.

Numerous studies have examined the amygdala volume in MDD; however, inconsistent even opposing results were obtained (Munn et al., 2007; Tang et al., 2007). Many factors may collectively account for the discrepancy, in which the clinical heterogeneity of the patients and the functional heterogeneity of the amygdala might be two key ones. Focusing on individual amygdala subregions in MDD patients with and without anxiety, we showed that there were no significant GMV differences in the patients compared with those in the HCs for any amygdala subregion. This is consistent with previous studies showing an unchanged amygdala volume in MDD (Bremner et al., 2000; Frodl et al., 2004; Munn et al., 2007; Lorenzetti et al., 2010). Moreover, no significant GMV differences were observed between the two patient groups for any amygdala subregion. This is in line with a previous study showing no significant volume differences of the whole amygdala between patients with anxious unipolar major depressive disorder and those with non-anxious unipolar major depressive disorder (Delaparte et al., 2017). These findings collectively suggest that gray matter volume of the amygdala is not a suitable sign to differentiate anxious depressed from non-anxious depressed individuals. Notably, there are a number of other variables that may confound results for the amygdala volume, including depression duration or number of episodes (Frodl et al., 2003; MacMaster et al., 2008; Kronenberg et al., 2009), antidepressant medication (Hamilton et al., 2008), suicidal tendency (Monkul et al., 2007; Spoletini et al., 2011), genetic variations (Zetzsche et al., 2008; Savitz and Drevets, 2009), and the state of MDD (van Eijndhoven et al., 2009; Malykhin et al., 2012; Zavorotnyy et al., 2018). Further studies are needed to examine the reproducibility of our findings by taking these factors into consideration to gain a more comprehensive picture of amygdala volume abnormalities in MDD. In addition, postmortem neuroanatomical studies may further clarify the issue by examining, for example, neuron or glia numbers or densities at the cellular level (Rubinow et al., 2016).

In addition to structural GMV, we characterized the functional organization of the amygdala subregions from the perspectives of local fluctuation amplitude, intra-region functional integration, and inter-regional functional communication. First, we found that patients with AMDD showed significantly decreased ALFF in the bilateral SFA and the LBA compared with those in the HCs. The SFA and LBA are closely related to emotional processing and regulation: The SFA is mainly involved in olfactory function (Heimer and Van Hoesen, 2006; Moreno and Gonzalez, 2007) and affective processes (Roy et al., 2009), and the LBA plays a central role in modulating the fear response and evaluating sensory information (Jovanovic and Ressler, 2010). In contrast, the CMA is mainly associated with the generation of behavioral responses through projections to the brainstem and cortical and striatal regions (Roy et al., 2009). Accordingly, our results suggest that AMDD selectively affects local spontaneous brain activity of emotion-related amygdala subregions. However, no significant ALFF differences were found between patients with nAMDD and HCs for any amygdala subregion. Therefore, the observed ALFF alterations in SFA and LBA appear to be specific to AMDD. This implies that the SFA and the LBA may be involved in the pathophysiological processes of AMDD and may serve as potential biomarkers to differentiate AMDD from nAMDD. However, no significant differences were found in the ALFF of the bilateral SFA and the LBA between the two patient groups. These findings imply that there may exist a trend effect in the observed ALFF alterations from nAMDD to AMDD. Notably, using a whole-brain voxel-wise analysis, two previous studies found increased ALFF in the bilateral amygdala in patients with MDD compared with that in HCs (Du et al., 2016; Chen et al., 2017). The discrepancy might be caused by different scales at which the analyses were performed (i.e., voxel-level vs. region-level) or because of the differentiation of AMDD and nAMDD in the current study. Moreover, we found that decreased ALFF in the left LBA and SFA were negatively correlated with the HAMD without anxiety/somatization factor scores in the AMDD patients. The HAMD without anxiety/somatization factor scores include several factors, such as cognitive disturbance, retardation, sleep disturbance, and weight loss. These factors represent different symptom clusters that are closely related to the characteristics and medication of the disease. Thus, the ALFF decreases may underlie the specific symptoms in patients with AMDD. Further studies are needed to deepen our understanding on this point by examining the associations between alterations of amygdala subregions and each factor in a larger sample size.

Second, no significant difference was found in the COSLOF among the three groups for each amygdala subregion. This suggests intact functional integration within each amygdala subregion in patients with MDD, regardless of the status of anxiety.

Finally, compared with the HCs, both patients with AMDD and those with nAMDD exhibited decreased functional connectivity of the bilateral SFA, with no significant differences between the two patient groups. This suggests that functional connectivity reductions of the SFA might serve as intrinsic features for MDD. Mounting evidence from both animal and human studies indicates that the SFA typically shows connectivity with the limbic system to form a neural circuit that is closely associated with affective processes (Moreno and Gonzalez, 2007; Goossens et al., 2009; Roy et al., 2009; Gabard-Durnam et al., 2014). Since affective disturbances are commonly observed in MDD, regardless of subtype, it is plausible to observe disrupted functional connectivity of the SFA in both AMDD and nAMDD. However, a previous task-fMRI study showed common deficits in both activation and connectivity of the amygdala among patients with MDD, patients with anxiety, and comorbid subjects, suggesting a shared origin between anxiety and depression (Etkin and Schatzberg, 2011). Specifically, the right SFA showed decreased functional connectivity with the bilateral ACC and ORBsupmed in both MDD groups. Both the ACC and ORBsupmed are parts of the prefrontal cortex and are related to emotional processing and regulation (Mayberg et al., 2002; Milad and Rauch, 2007). Numerous previous studies demonstrated the existence of functional connectivity between the amygdala and different parts of the prefrontal regions (e.g., the ACC, and the ventral prefrontal and orbitofrontal cortex) in healthy individuals (Zald and Pardo, 1997; Hariri et al., 2003; Ochsner et al., 2004) and disruptions of these connectivities in MDD (Almeida et al., 2009; Versace et al., 2010; Etkin and Schatzberg, 2011). The disruptions imply impaired prefrontal modulation of the amygdala in MDD, which may contribute to emotional disturbances in patients suffering from the disease. Here, our findings further suggested that the disruptions are selectively targeted to the SFA in MDD. As for the left SFA, decreased functional connectivity with the left putamen was found in both MDD groups, as compared with that in the HCs. The putamen belongs to the dorsal striatum and is thought to play a crucial role in the development of mood disorders. For instance, one animal study found that the putamen was significantly different between anhedonic-like and resilient animals, indicating important implications of the putamen in anhedonia (Delgado y Palacios et al., 2014), which is one of the core symptoms of depression. Thus, the disrupted interaction between the left SFA and the putamen may be involved in the pathological mechanism of depression, in particular the origin of anhedonia. It should be noted that to what extent the findings from animal studies apply to humans remain to be determined due to the limitation of cross-species homologies. This issue can be partially addressed in the future by establishing cross-species correspondence in brain structures via network approaches (Goulas et al., 2015).

Several limitations of this study should be mentioned. First, the sample size was relatively small, which may limit the generalizability of our findings. Second, about half of the patients were treated with psychotropic medication before the MRI scan, which may have significantly modulated their functional brain organization (Wang et al., 2015c; Abdallah et al., 2017; Sheng et al., 2018). Although all patients were free of psychotropic medications for at least 4 weeks before the MRI scan, we cannot fully exclude the possible long-term effects of medication. Studies on non-medicated patients are expected in the future to examine to what extent the current findings are contaminated by this factor. Third, similar to previous studies, we employed dimensional diagnosis to define AMDD, based on the severity of anxiety symptoms in MDD, as measured by the HAMD anxiety/somatization factor. However, the anxiety/somatization factor only contains seven items representing anxiety symptoms and thus may suffer from the risk of misclassification to some extent. Nevertheless, it should be noted that a previous study in Level 1 of the Sequenced Treatment Alternatives to Relieve Depression indicated that the association between anxious unipolar major depressive disorder and poorer treatment outcome was independent of how we defined anxious unipolar major depressive disorder (Fava et al., 2008). Future studies are required to replicate the current findings by integrating multiple dimension diagnosis to minimize the classification error. Fourth, although spatial smoothing was performed after masking out all amygdala subregions to ensure functional specificity, the signals in the amygdala subregions might still be polluted by those from regions adjacent to them. Future studies are required to address this issue by using more sophisticated methods, such as orthogonalization. Finally, several previous studies have shown that MDD-related alterations in both local fluctuation amplitude and interregional functional connectivity are frequency-dependent (Yue et al., 2015; He et al., 2016; Wang et al., 2016). It would be interesting to investigate whether the observed amygdala alterations are modulated by choices of different frequency intervals.

## Conclusion

In summary, we demonstrated common and specific functional alterations of the amygdala between patients with AMDD and those with nAMDD. Moreover, the alterations were dependent on the functional features chosen and different amygdala subregions. These findings deepen our understanding neural mechanisms underlying anxiety co-morbid with MDD and have implications for future development of new and effective treatment programs.

## Data Availability Statement

The original contributions presented in the study are included in the article/supplementary material, further inquiries can be directed to the corresponding authors.

## Ethics Statement

The studies involving human participants were reviewed and approved by The ethics committee of Sir Run Run Shaw Hospital, Zhejiang University School of Medicine. The patients/participants provided their written informed consent to participate in this study.

## Author Contributions

YL, XN, and YY conducted the statistical analysis and drafted the initial manuscript. YQ, PW, JY, KR, LZ, ZL, and TS contributed to the conduct of the study. JW conducted the data analysis. Y-FZ and JW were responsible for the interpretation of MRI data. WC and JW reviewed and revised the manuscript. WC and YS designed and supervised the study. All authors contributed to the article and approved the submitted version.

## Conflict of Interest

The authors declare that the research was conducted in the absence of any commercial or financial relationships that could be construed as a potential conflict of interest.

## References

[B1] Abdallah C. G. Averill L. A. Collins K. A. Geha P. Schwartz J. Averill C.. (2017). Ketamine treatment and global brain connectivity in major depression. Neuropsychopharmacology 42, 1210–1219. 10.1038/npp.2016.186 27604566 PMC5437875

[B2] Almeida J. R. Versace A. Mechelli A. Hassel S. Quevedo K. Kupfer D. J.. (2009). Abnormal amygdala-prefrontal effective connectivity to happy faces differentiates bipolar from major depression. Biol. Psychiatry 66, 451–459. 10.1016/j.biopsych.2009.03.024 19450794 PMC2740996

[B3] Amunts K. Kedo O. Kindler M. Pieperhoff P. Mohlberg H. Shah N. J.. (2005). Cytoarchitectonic mapping of the human amygdala, hippocampal region, and entorhinal cortex: intersubject variability and probability maps. Anat. Embryol. (Berl.) 210, 343–352. 10.1007/s00429-005-0025-5 16208455

[B4] Ball T. Rahm B. Eickhoff S. B. Schulze-Bonhage A. Speck O. Mutschler I. (2007). Response properties of human amygdala subregions: evidence based on functional MRI combined with probabilistic anatomical maps. PLoS ONE 2:e307. 10.1371/journal.pone.0000307 17375193 PMC1819558

[B5] Bremner J. D. Narayan M. Anderson E. R. Staib L. H. Miller H. L. Charney D. S. (2000). Hippocampal volume reduction in major depression. Am. J. Psychiatry 157, 115–118. 10.1176/ajp.157.1.115 10618023

[B6] Chen V. C. Shen C. Y. Liang S. H. Li Z. H. Hsieh M. H. Tyan Y. S.. (2017). Assessment of brain functional connectome alternations and correlation with depression and anxiety in major depressive disorders. PeerJ. 5:e3147. 10.7717/peerj.3147 29181274 PMC5702252

[B7] Delaparte L. Yeh F. C. Adams P. Malchow A. Trivedi M. H. Oquendo M. A.. (2017). A comparison of structural connectivity in anxious depression versus non-anxious depression. J. Psychiatr. Res. 89, 38–47. 10.1016/j.jpsychires.2017.01.012 28157545 PMC5374003

[B8] Delgado y Palacios R. Verhoye M. Henningsen K. Wiborg O. Van der Linden A. (2014). Diffusion kurtosis imaging and high-resolution MRI demonstrate structural aberrations of caudate putamen and amygdala after chronic mild stress. PLoS ONE 9:e95077. 10.1371/journal.pone.0095077 24740310 PMC3989315

[B9] Du L. Wang J. Meng B. Yong N. Yang X. Huang Q.. (2016). Early life stress affects limited regional brain activity in depression. Sci. Rep. 6:25338. 10.1038/srep25338 27138376 PMC4853783

[B10] Etkin A. Schatzberg A. F. (2011). Common abnormalities and disorder-specific compensation during implicit regulation of emotional processing in generalized anxiety and major depressive disorders. Am. J. Psychiatry 168, 968–978. 10.1176/appi.ajp.2011.10091290 21632648

[B11] Fava M. Rush A. J. Alpert J. E. Balasubramani G. K. Wisniewski S. R. Carmin C. N.. (2008). Difference in treatment outcome in outpatients with anxious versus nonanxious depression: a STAR^*^D report. Am. J. Psychiatry 165, 342–351. 10.1176/appi.ajp.2007.06111868 18172020

[B12] Fonseka T. M. MacQueen G. M. Kennedy S. H. (2018). Neuroimaging biomarkers as predictors of treatment outcome in Major Depressive Disorder. J. Affect. Disord. 233, 21–35. 10.1016/j.jad.2017.10.049 29150145

[B13] Friston K. J. Williams S. Howard R. Frackowiak R. S. Turner R. (1996). Movement-related effects in fMRI time-series. Magn. Reson. Med. 35, 346–355. 10.1002/mrm.1910350312 8699946

[B14] Frodl T. Meisenzahl E. M. Zetzsche T. Born C. Jager M. Groll C.. (2003). Larger amygdala volumes in first depressive episode as compared to recurrent major depression and healthy control subjects. Biol. Psychiatry 53, 338–344. 10.1016/S0006-3223(02)01474-9 12586453

[B15] Frodl T. Meisenzahl E. M. Zetzsche T. Höhne T. Banac S. Schorr C.. (2004). Hippocampal and amygdala changes in patients with major depressive disorder and healthy controls during a 1-year follow-up. J. Clin. Psychiatry 65, 492–499. 10.4088/JCP.v65n0407 15119911

[B16] Gabard-Durnam L. J. Flannery J. Goff B. Gee D. G. Humphreys K. L. Telzer E.. (2014). The development of human amygdala functional connectivity at rest from 4 to 23 years: a cross-sectional study. Neuroimage 95, 193–207. 10.1016/j.neuroimage.2014.03.038 24662579 PMC4305511

[B17] Gaspersz R. Lamers F. Kent J. M. Beekman A. T. Smit J. H. van Hemert A. M.. (2017a). Longitudinal predictive validity of the DSM-5 anxious distress specifier for clinical outcomes in a large cohort of patients with major depressive disorder. J. Clin. Psychiatry 78, 207–213. 10.4088/JCP.15m10221 27035515

[B18] Gaspersz R. Lamers F. Kent J. M. Beekman A. T. F. Smit J. H. van Hemert A. M.. (2017b). Anxious distress predicts subsequent treatment outcome and side effects in depressed patients starting antidepressant treatment. J. Psychiatr. Res. 84, 41–48. 10.1016/j.jpsychires.2016.09.018 27693981

[B19] Goldberg D. Fawcett J. (2012). The importance of anxiety in both major depression and bipolar disorder. Depress Anxiety 29, 471–478. 10.1002/da.21939 22553107

[B20] Goldberg D. P. Wittchen H. U. Zimmermann P. Pfister H. Beesdobaum K. (2014). Anxious and non-anxious forms of major depression: familial, personality, and symptom characteristics. Psychol. Med. 44:1223. 10.1017/S0033291713001827 23902895

[B21] Goossens L. Kukolja J. Onur O. A. Fink G. R. Maier W. Griez E.. (2009). Selective processing of social stimuli in the superficial amygdala. Hum. Brain Mapp. 30, 3332–3338. 10.1002/hbm.20755 19347877 PMC6870612

[B22] Goulas A. Schaefer A. Margulies D. S. (2015). The strength of weak connections in the macaque cortico-cortical network. Brain Struct. Funct. 220, 2939–2951. 10.1007/s00429-014-0836-3 25035063

[B23] Hallquist M. N. Hwang K. Luna B. The nuisance of nuisance regression: spectral misspecification in a common approach to resting-state fMRI preprocessing reintroduces noise and obscures functional connectivity. Neuroimage. (2013) 82, 208–25. 10.1016/j.neuroimage.2013.05.116 23747457 PMC3759585

[B24] Hamilton J. P. Siemer M. Gotlib I. H. (2008). Amygdala volume in major depressive disorder: a meta-analysis of magnetic resonance imaging studies. Mol. Psychiatry 13, 993–1000. 10.1038/mp.2008.57 18504424 PMC2739676

[B25] Hariri A. R. Mattay V. S. Tessitore A. Fera F. Weinberger D. R. (2003). Neocortical modulation of the amygdala response to fearful stimuli. Biol. Psychiatry 53, 494–501. 10.1016/S0006-3223(02)01786-9 12644354

[B26] He Z. Cui Q. Zheng J. Duan X. Pang Y. Gao Q.. (2016). Frequency-specific alterations in functional connectivity in treatment-resistant and -sensitive major depressive disorder. J. Psychiatr. Res. 82, 30–39. 10.1016/j.jpsychires.2016.07.011 27459030

[B27] Heimer L. Van Hoesen G. W. (2006). The limbic lobe and its output channels: implications for emotional functions and adaptive behavior. Neurosci. Biobehav. Rev. 30, 126–147. 10.1016/j.neubiorev.2005.06.006 16183121

[B28] Ionescu D. F. Niciu M. J. Richards E. M. Zarate C. A. Jr. (2014). Pharmacologic treatment of dimensional anxious depression: a review. Prim. Care Companion CNS Disord. 16:PCC.13r01621. 10.4088/PCC.13r01621 25317369 PMC4195641

[B29] Jovanovic T. Ressler K. J. (2010). How the neurocircuitry and genetics of fear inhibition may inform our understanding of PTSD. Am. J. Psychiatry 167, 648–662. 10.1176/appi.ajp.2009.09071074 20231322 PMC3603297

[B30] Kronenberg G. Tebartz van Elst L. Regen F. Deuschle M. Heuser I. Colla M. (2009). Reduced amygdala volume in newly admitted psychiatric in-patients with unipolar major depression. J. Psychiatr. Res. 43, 1112–1117. 10.1016/j.jpsychires.2009.03.007 19394960

[B31] Li S. J. Li Z. Wu G. Zhang M. J. Franczak M. Antuono P. G. (2002). Alzheimer disease: evaluation of a functional MR imaging index as a marker. Radiology 225, 253–259. 10.1148/radiol.2251011301 12355013

[B32] Li Y. Qin W. Jiang T. Zhang Y. Yu C. (2012). Sex-dependent correlations between the personality dimension of harm avoidance and the resting-state functional connectivity of amygdala subregions. PLoS ONE 7:e35925. 10.1371/journal.pone.0035925 22558274 PMC3338761

[B33] Licznerski P. Duman R. S. (2013). Remodeling of axo-spinous synapses in the pathophysiology and treatment of depression. Neuroscience 251, 33–50. 10.1016/j.neuroscience.2012.09.057 23036622 PMC3566360

[B34] Lorenzetti V. Allen N. B. Whittle S. Yücel M. (2010). Amygdala volumes in a sample of current depressed and remitted depressed patients and healthy controls. J. Affect. Disord. 120, 112–119. 10.1016/j.jad.2009.04.021 19464062

[B35] MacMaster F. P. Mirza Y. Szeszko P. R. Kmiecik L. E. Easter P. C. Taormina S. P.. (2008). Amygdala and hippocampal volumes in familial early onset major depressive disorder. Biol. Psychiatry 63, 385–390. 10.1016/j.biopsych.2007.05.005 17640621 PMC2268763

[B36] Malykhin N. V. Carter R. Hegadoren K. M. Seres P. Coupland N. J. (2012). Fronto-limbic volumetric changes in major depressive disorder. J. Affect. Disord. 136, 1104–1113. 10.1016/j.jad.2011.10.038 22134041

[B37] Mayberg H. S. Silva J. A. Brannan S. K. Tekell J. L. Mahurin R. K. McGinnis S.. (2002). The functional neuroanatomy of the placebo effect. Am. J. Psychiatry 159, 728–737. 10.1176/appi.ajp.159.5.728 11986125

[B38] Milad M. R. Rauch S. L. (2007). The role of the orbitofrontal cortex in anxiety disorders. Ann. N. Y. Acad. Sci. 1121, 546–561. 10.1196/annals.1401.006 17698998

[B39] Monkul E. S. Hatch J. P. Nicoletti M. A. Spence S. Brambilla P. Lacerda A. L.. (2007). Fronto-limbic brain structures in suicidal and non-suicidal female patients with major depressive disorder. Mol. Psychiatry 12, 360–366. 10.1038/sj.mp.4001919 17389903

[B40] Moreno N. Gonzalez A. (2007). Evolution of the amygdaloid complex in vertebrates, with special reference to the anamnio-amniotic transition. J. Anat. 211, 151–163. 10.1111/j.1469-7580.2007.00780.x 17634058 PMC2375767

[B41] Munn M. A. Alexopoulos J. Nishino T. Babb C. M. Flake L. A. Singer T.. (2007). Amygdala volume analysis in female twins with major depression. Biol. Psychiatry 62, 415–422. 10.1016/j.biopsych.2006.11.031 17511971 PMC2904677

[B42] Ochsner K. N. Ray R. D. Cooper J. C. Robertson E. R. Chopra S. Gabrieli J. D.. (2004). For better or for worse: neural systems supporting the cognitive down- and up-regulation of negative emotion. Neuroimage 23, 483–499. 10.1016/j.neuroimage.2004.06.030 15488398

[B43] Ongur D. Price J. L. (2000). The organization of networks within the orbital and medial prefrontal cortex of rats, monkeys and humans. Cereb. Cortex 10, 206–219. 10.1093/cercor/10.3.206 10731217

[B44] Phillips M. L. Swartz H. A. (2014). A critical appraisal of neuroimaging studies of bipolar disorder: toward a new conceptualization of underlying neural circuitry and a road map for future research. Am. J. Psychiatry. 171, 829–843. 10.1176/appi.ajp.2014.13081008 24626773 PMC4119497

[B45] Qin S. Young C. B. Duan X. Chen T. Supekar K. Menon V. (2014). Amygdala subregional structure and intrinsic functional connectivity predicts individual differences in anxiety during early childhood. Biol. Psychiatry 75, 892–900. 10.1016/j.biopsych.2013.10.006 24268662 PMC3984386

[B46] Rao S. Zisook S. (2009). Anxious depression: clinical features and treatment. Curr. Psychiatry Rep. 11, 429–436. 10.1007/s11920-009-0065-2 19909663

[B47] Roy A. K. Shehzad Z. Margulies D. S. Kelly A. M. Uddin L. Q. Gotimer K.. (2009). Functional connectivity of the human amygdala using resting state fMRI. Neuroimage 45, 614–626. 10.1016/j.neuroimage.2008.11.030 19110061 PMC2735022

[B48] Rubinow M. J. Mahajan G. May W. Overholser J. C. Jurjus G. J. Dieter L.. (2016). Basolateral amygdala volume and cell numbers in major depressive disorder: a postmortem stereological study. Brain Struct. Funct. 221, 171–184. 10.1007/s00429-014-0900-z 25287512 PMC4388764

[B49] Savitz J. B. Drevets W. C. (2009). Imaging phenotypes of major depressive disorder: genetic correlates. Neuroscience 164, 300–330. 10.1016/j.neuroscience.2009.03.082 19358877 PMC2760612

[B50] Seo H. J. Jung Y. E. Kim T. S. Kim J. B. Lee M. S. Kim J. M.. (2011). Distinctive clinical characteristics and suicidal tendencies of patients with anxious depression. J. Nerv. Ment. Dis. 199, 42–48. 10.1097/NMD.0b013e3182043b60 21206246

[B51] Shen Y. Yao J. Jiang X. Zhang L. Xu L. Feng R.. (2015). Sub-hubs of baseline functional brain networks are related to early improvement following two-week pharmacological therapy for major depressive disorder. Hum. Brain Mapp. 36, 2915–2927. 10.1002/hbm.22817 25930660 PMC6869049

[B52] Sheng J. Shen Y. Qin Y. Zhang L. Jiang B. Li Y.. (2018). Spatiotemporal, metabolic, and therapeutic characterization of altered functional connectivity in major depressive disorder. Hum. Brain Mapp. 39, 1957–1971. 10.1002/hbm.23976 29341320 PMC6866283

[B53] Spoletini I. Piras F. Fagioli S. Rubino I. A. Martinotti G. Siracusano A.. (2011). Suicidal attempts and increased right amygdala volume in schizophrenia. Schizophr. Res. 125, 30–40. 10.1016/j.schres.2010.08.023 20869847

[B54] Tang Y. Wang F. Xie G. Liu J. Li L. Su L.. (2007). Reduced ventral anterior cingulate and amygdala volumes in medication-naive females with major depressive disorder: a voxel-based morphometric magnetic resonance imaging study. Psychiatry Res. 156, 83–86. 10.1016/j.pscychresns.2007.03.005 17825533

[B55] van Eijndhoven P. van Wingen G. van Oijen K. Rijpkema M. Goraj B. Jan Verkes R.. (2009). Amygdala volume marks the acute state in the early course of depression. Biol. Psychiatry 65, 812–818. 10.1016/j.biopsych.2008.10.027 19028381

[B56] van Tol M. J. Demenescu L. R. van der Wee N. J. Kortekaas R. Marjan M. A. N. Boer J. A.. (2012). Functional magnetic resonance imaging correlates of emotional word encoding and recognition in depression and anxiety disorders. Biol. Psychiatry 71, 593–602. 10.1016/j.biopsych.2011.11.016 22206877

[B57] Versace A. Thompson W. K. Zhou D. Almeida J. R. Hassel S. Klein C. R.. (2010). Abnormal left and right amygdala-orbitofrontal cortical functional connectivity to emotional faces: state versus trait vulnerability markers of depression in bipolar disorder. Biol. Psychiatry 67, 422–431. 10.1016/j.biopsych.2009.11.025 20159144 PMC2835157

[B58] Wang J. Wang X. He Y. Yu X. Wang H. He Y. (2015a). Apolipoprotein E ε4 modulates functional brain connectome in Alzheimer's disease. Hum. Brain Mapp. 36, 1828–1846. 10.1002/hbm.22740 25619771 PMC6869368

[B59] Wang J. Wang X. Xia M. Liao X. Evans A. He Y. (2015b). GRETNA: a graph theoretical network analysis toolbox for imaging connectomics. Front. Hum. Neurosci. 9:386. 10.3389/fnhum.2015.00386 26175682 PMC4485071

[B60] Wang J. Wei Q. Bai T. Zhou X. Sun H. Becker B.. (2017). Electroconvulsive therapy selectively enhanced feedforward connectivity from fusiform face area to amygdala in major depressive disorder. Soc. Cogn. Affect. Neurosci. 12, 1983–1992. 10.1093/scan/nsx100 28981882 PMC5716231

[B61] Wang L. Kong Q. Li K. Su Y. Zeng Y. Zhang Q.. (2016). Frequency-dependent changes in amplitude of low-frequency oscillations in depression: a resting-state fMRI study. Neurosci. Lett. 614, 105–111. 10.1016/j.neulet.2016.01.012 26797652

[B62] Wang L. Xia M. Li K. Zeng Y. Su Y. Dai W.. (2015c). The effects of antidepressant treatment on resting-state functional brain networks in patients with major depressive disorder. Hum. Brain Mapp. 36, 768–778. 10.1002/hbm.22663 25332057 PMC6869500

[B63] Wiethoff K. Bauer M. Baghai T. C. Möller H. J. Fisher R. Hollinde D.. (2010). Prevalence and treatment outcome in anxious versus nonanxious depression: results from the German Algorithm Project. J. Clin. Psychiatry 71, 1047–1054. 10.4088/JCP.09m05650blu 20673545

[B64] Yue Y. Jia X. Hou Z. Zang Y. Yuan Y. (2015). Frequency-dependent amplitude alterations of resting-state spontaneous fluctuations in late-onset depression. Biomed. Res. Int. 2015:505479. 10.1155/2015/505479 25705666 PMC4331395

[B65] Zald D. H. Pardo J. V. (1997). Emotion, olfaction, and the human amygdala: amygdala activation during aversive olfactory stimulation. Proc. Natl. Acad. Sci. U.S.A. 94, 4119–4124. 10.1073/pnas.94.8.4119 9108115 PMC20578

[B66] Zang Y. F. He Y. Zhu C. Z. Cao Q. J. Sui M. Q. Liang M.. (2007). Altered baseline brain activity in children with ADHD revealed by resting-state functional MRI. Brain Dev. 29, 83–91. 10.1016/j.braindev.2006.07.002 16919409

[B67] Zavorotnyy M. Zollner R. Schulte-Gustenberg L. R. Wulff L. Schoning S. Dannlowski U.. (2018). Low left amygdala volume is associated with a longer duration of unipolar depression. J. Neural. Transm. (Vienna) 125, 229–238. 10.1007/s00702-017-1811-y 29159580

[B68] Zetzsche T. Preuss U. W. Bondy B. Frodl T. Zill P. Schmitt G.. (2008). 5-HT1A receptor gene C−1019 G polymorphism and amygdala volume in borderline personality disorder. Genes Brain Behav. 7, 306–313. 10.1111/j.1601-183X.2007.00353.x 18387137

